# Case report: A novel mutation of RecQ-like helicase 5 in a Chinese family with early myocardial infarction, coronary artery disease, and stroke hemiplegia

**DOI:** 10.3389/fgene.2023.1146932

**Published:** 2023-04-26

**Authors:** Yi Tang, Qian Wang, Wei-Kai Zhang, Yu-Xing Liu, Zhao-Fen Zheng, Liang-Liang Fan, Lv Liu, Jin He

**Affiliations:** ^1^Department of Cardiology, Hunan Provincial People’s Hospital, The First Affiliated Hospital of Hunan Normal University, Clinical Medicine Research Center of Heart Failure of Hunan Province, Hunan Normal University, Changsha, China; ^2^Department of Cell Biology, School of Life Sciences, Central South University, Changsha, China; ^3^Department of Respiratory Medicine, Diagnosis and Treatment Center of Respiratory Disease, The Second Xiangya Hospital of Central South University, Changsha, China

**Keywords:** myocardial infarction, coronary artery disease, stroke hemiplegia, RECQL5, whole-exome sequencing, novel mutation

## Abstract

**Background:** Myocardial infarction (MI) is a type of severe coronary artery disease (CAD) that can lead to heart failure and sudden cardiac death. The prevalence of heart failure globally is estimated at 1%–2%, of which ∼60% of cases are the consequence of MI as the primary cause. At present, several disease-causing genes have been identified that may be responsible for MI, such as autophagy-related 16-like 1 (ATG16L1) and RecQ-like helicase 5 (RECQL5).

**Methods:** In this study, we enrolled a Chinese family with MI, CAD, and stroke hemiplegia. Whole-exome sequencing was applied to analyze the genetic lesion of the proband. Sanger sequencing was used to validate the candidate mutation in five family members and 200 local control cohorts.

**Results:** After data filtering, we detected a novel mutation (NM_004259: c.1247T>C/p.I416T) of RECQL5 in the proband. Sanger sequencing further validated that the novel mutation was existent in the affected individuals, including the proband’s younger sister and her mother, and absent in the other healthy family members and 200 local control cohorts. Furthermore, bioinformatics analysis confirmed that the novel mutation, located in a highly evolutionarily conserved site, was predicted to be deleterious and may change the hydrophobic surface area and aliphatic index of RECQL5.

**Conclusion:** Here, we report the second mutation (NM_004259: c.1247T>C/p.I416T) of RECQL5 underlying MI and CAD by whole-exome sequencing. Our study expanded the spectrum of RECQL5 mutations and contributed to genetic diagnosis and counseling of MI and CAD.

## Introduction

Myocardial infarction (MI) results from disruption of a vulnerable atherosclerotic plaque or erosion of the coronary artery endothelium. The typical symptom of MI is coronary blood flow cut off, which can lead to critical myocardial necrosis, further causing heart failure or cardiac rupture, and some critical patients progress to cardiac death (Reed et al., 2017; Kaier et al., 2021). The prevalence of heart failure globally is estimated at 1%–2%, of which ∼60% of cases are the consequence of MI as the primary cause (Saleh and Ambrose, 2018; Kaier et al., 2021). MI has been recognized as the most rapidly increasing contributor to the global burden of disease (Salwan and Seth, 2021).

In addition to the environmental factors, genetic factors play a crucial role in MI. Multiple genome-wide association studies identified novel loci and candidate genes in MI, such as 9p21.3 and 1p13.3 (Haver et al., 2014). Recent studies have shown that some lipid metabolism genes, such as cholesteryl ester transfer protein (CETP) and ATP-binding cassette subfamily A member 1 (ABCA1) may indirectly cause MI by affecting coronary atherosclerosis progression (Li et al., 2021; Wang et al., 2022). Simultaneously, mutations in 1-acylglycerol-3-phosphate-O-acyltransferase 2 (AGPAT2), autophagy-related 16-like 1 (ATG16L1), and RecQ-like helicase 5 (RECQL5) have been reported to possibly lead to MI in patients (Xie et al., 2016; Montenegro Junior et al., 2020; Han et al., 2021). In addition, some correlation studies imply that polymorphisms in energy metabolism-related genes such as mitochondrially encoded tRNA leucine 1 (MT-TL1) and isocitrate dehydrogenase (NADP(+)) 2 (IDH2) may be associated with the onset of MI (Cosma et al., 2022; Watany et al., 2022).

Here, we enrolled a Chinese family with MI, coronary artery disease (CAD), and stroke hemiplegia. Whole-exome sequencing and Sanger sequencing were applied to explore the genetic lesion of the affected individuals.

## Case presentation

**FIGURE 1:**
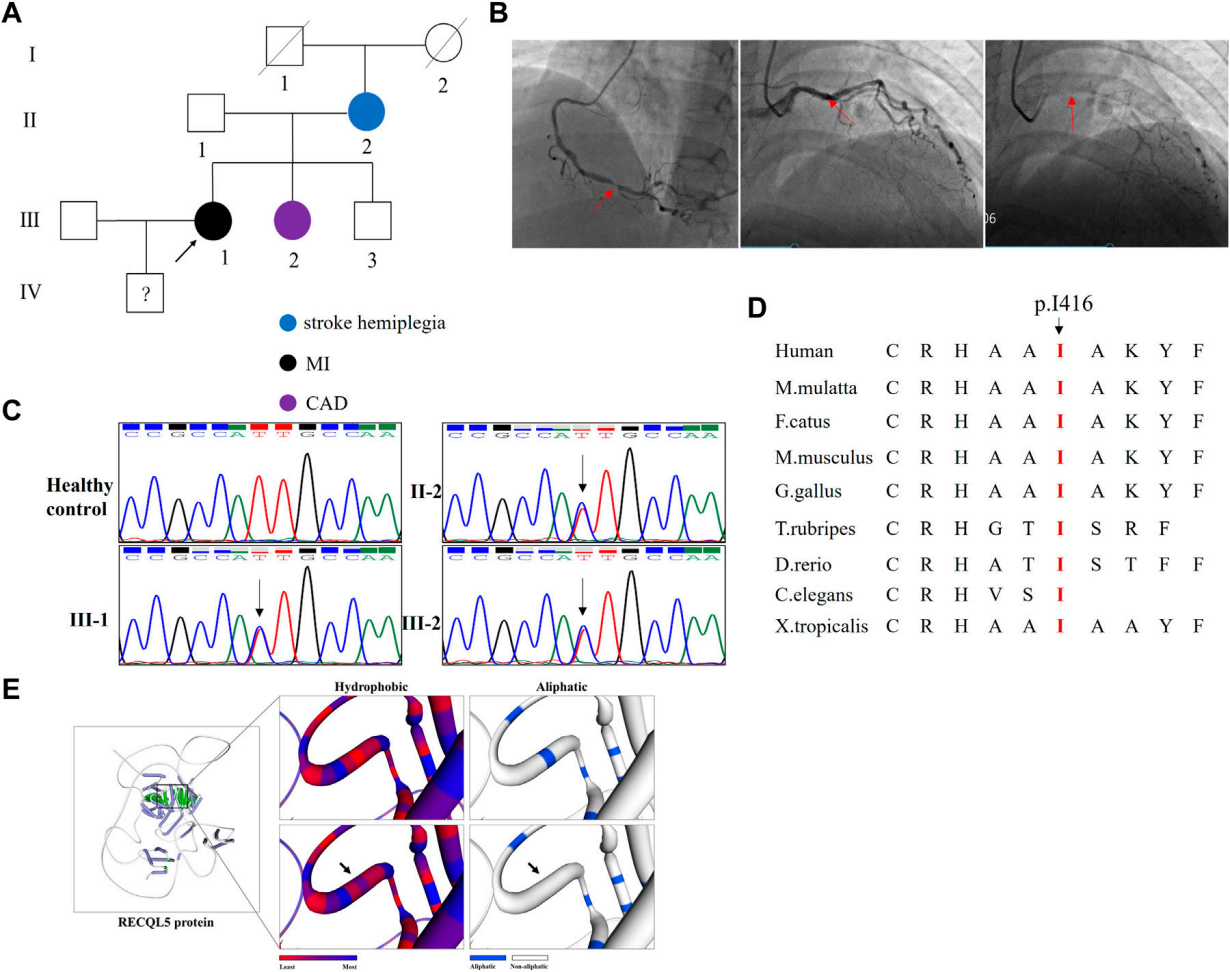
Clinical data and genetic analysis of the family. **(A)** Pedigree of the family. White circles/squares denote the unaffected family members, and the arrow indicates the proband. **(B)** Coronary angiography presented the right coronary stenosis (first picture) and left coronary stenosis (the second and third pictures). **(C)** Sanger sequencing of RECQL5 confirmed the novel mutation (NM_004259: c.1247T>C/p.I416T) in the proband and her affected sister. The arrows indicate the mutation site. **(D)** Alignment of multiple RECQL5 protein sequences across species. The I416-affected amino acid locates in the highly conserved amino acid region in different mammals (from Ensembl). The red column shows the I416 site. **(E)** The wild-type RECQL5 (WT) protein structure and the mutant RECQL5 (p.I416T) protein structure were predicted by SWISS-MODEL online software. The hydrophobic surface area and aliphatic index of the WT and mutated RECQL5 were predicted.

In this study, we enrolled a family from Hunan Province, China (Figure 1A; Table 1). The proband, a 45-year-old woman, was admitted to our hospital due to recurrent chest congestion and chest pain for 2 months, aggravated for 1 day. The troponin I level was 0.078 microgram/L (normal reference value, 0–0.023 microgram/L), and those of creatine kinase (54.0 U/L) and creatine kinase isoenzyme MB (14.2 U/L) were higher than normal levels. Coronary angiography suggested stenosis of multiple coronary arteries and complete occlusion of the left anterior descending coronary artery (Figure 1B). Finally, the patient was diagnosed with non-ST segment elevation myocardial infarction. Medical history investigation revealed that she also suffered from hypertension and hyperuricemia (551 μmol/L) and had experienced cerebral infarction 2 years ago. A survey of the family history suggested that one of her sisters (III-2) also suffered from CAD and her mother (II-2) suffered from stroke hemiplegia.

**TABLE 1:** Clinical characteristics of the affected family members.

Sample	Age (Y)	CHOL (mmol/L)	CLU (mmol/L)	HDLC (mmol/L)	LDLC (mmol/L)	TG (mmol/L)	URIC (mmol/L)	CRP (mg/L)	BMI (kg/m^2^)
II-2	66	6.62	6.22	0.32	3.41	3.15	530	45.6	23.4
III-1	45	7.13	7.39	0.75	4.03	2.28	551	21.4	22.7
III-2	42	6.78	6.07	0.46	3.95	3.33	447	19.7	24.5

BMI, body mass index; CHOL, cholesterol; CRP, C-reactive protein; GLU, glucose; HDLC, high-density lipoprotein; LDL, low-density lipoprotein; URIC, uric acid.

## Laboratory investigations

Genomic DNA was extracted from peripheral blood lymphocytes of all the five family members with a DNeasy Blood and Tissue Kit (Qiagen, Valencia, CA) following the manufacturer’s instruction. The proband (III-1) was selected to perform the whole-exome sequencing. Exome capture and next-generation sequencing were conducted by Berry Genomics Biotech Company (Beijing, China). The strategies of data filtering are as follows (Yu et al., 2023): (a) non-synonymous SNPs or frameshift-causing INDELs with an alternative allele frequency >0.05 in the NHLBI Exome Sequencing Project Exome Variant Server (ESP6500), dbSNP152 (http://www.ncbi.nlm.nih.gov/projects/SNP/index.html), the 1000 Genomes Project (http://www.1000genomes.org/), the ExAC database (http://exac.broadinstitute.org), or in-house exome databases of Berry Genomics (2000 exomes) were excluded; (b) the filtered SNVs and INDELs, predicted by SIFT (http://sift.jcvi.org/), PolyPhen2 (http://genetics.bwh.harvard.edu/pph2/), and MutationTaster (http://www.mutationtaster.org/) to be damaging, were retained; (c) co-segregation analysis was conducted on the family members by Sanger sequencing.

Whole-exome sequencing yielded 9.84 Gb data with 98.9% coverage of the target region and 97.2% of the target covered over 10×. After alignment and single-nucleotide variant calling, 80,728 variants were identified in the proband. Then, via the aforementioned filtering method and Sanger sequencing validation, a novel mutation (NM_004259: c.1247T>C/p.I416T) of RECQL5 was identified in the proband. No other known gene mutation underlying CAD, MI, hypertension, hyperuricemia, and hemiplegia was detected. Additional Sanger analysis confirmed that the novel mutation was existent in the affected siblings and absent in the healthy controls and 200 local control cohorts (Figure 1C). This mutation was also absent in public databases such as the 1000 Genomes Project, gnomAD, and Exome Aggregation Consortium (ExAC). Cross-species alignment analysis of RECQL5 amino acid sequences revealed that this mutated site was highly evolutionarily conserved (Figure 1D). SWISS-MODEL online software revealed that the p. I416T mutation changed the hydrophobic surface area and aliphatic index of RECQL5 (Figure 1E).

## Discussion

RECQL5, a member of the RecQ helicase family, plays an important role in replication, transcription, and especially repair. The RecQ family of helicases represents one of the most highly conserved groups of 3′-5′ DNA helicases and is named after the prototype *Escherichia coli* RecQ, called RECQL1, BLM, WRN, RECQL4, and RECQL5. Biallelic mutations in these RecQ homologs, WRN, BLM, and RECQL4, have been previously reported to be associated with rare human genetic diseases characterized by chromosomal instability and cancer susceptibility (Croteau et al., 2014; Thakkar et al., 2022). In 2022, Abu-Libdeh et al. reported that RECQL1 mutations can induce impaired DNA damage repair ability and lead to RECON syndrome, a genome instability disorder (Abu-Libdeh et al., 2022). RECQL5 deficiency may disrupt RAD51 recombinase nucleoprotein filaments and lead to genome instability (Hu et al., 2007; Andrs et al., 2020). Several studies have also indicated that RECQL5 was involved in multiple hereditary cancer susceptibility (Tavera-Tapia et al., 2019; Xia et al., 2021; Thakkar et al., 2022).

At present, only one study reported that RECQL5 mutations may result in MI and CAD (Xie et al., 2016). The study by Izumikawa et al. also revealed that RECQL5 may regulate the expression of LDLR, an essential gene for the regulation of plasma cholesterol, which was associated with MI and CAD (Izumikawa et al., 2008). In addition, knocking down the expression of RECQL5 may also affect the expression of β-actin, another gene related to cardiac remodeling and MI (Izumikawa et al., 2008). Here, in our study, the family members who carried the novel mutation (p.I416T) presented different clinical features. However, the MI, CAD, and stroke clinical features can result from the increase of plasma cholesterol, which was regulated by RECQL5. The different phenotypes may be caused by environmental factors and genetic heterogeneity.

The human RECQL5 gene, located on 17q25.1, encodes a DNA helicase named RecQ-like helicase 5, which contains a helicase domain, a RECQC domain, and a long C-terminal with an NLS domain (Saponaro et al., 2014). The mutation (NM_004259: c.1247T>C/p.I416T) is located in the helicase domain, which contains a conserved Zn^2+^-binding subdomain essential for efficient helicase activity (Newman et al., 2017). Hence, according to the SWISS-MODEL analysis, the mutation may affect the hydrophobic surface area and aliphatic index of RECQL5 and finally reduce the helicase activity and induce MI and CAD.

## Conclusion

In summary, we identified a novel mutation (NM_004259: c.1247T>C/p.I416T) of RECQL5 in a Han-Chinese family with MI, CAD, and stroke hemiplegia by whole-exome sequencing and Sanger sequencing. We reported the second mutation of RECQL5 underlying MI and CAD. Our study expanded the spectrum of RECQL5 mutations and contributed to genetic diagnosis and counseling of MI and CAD.

## Data Availability

The datasets presented in this study can be found in online repositories. The names of therepository/repositories and accession number(s) can be found below: https://ngdc.cncb.ac.cn/gsa-human/browse/HRA004190, China National Center for Bioinformation (HRA004190).

## References

[B1] Abu-Libdeh B. Jhujh S. S. Dhar S. Sommers J. A. Datta A. Longo G. M. (2022). RECON syndrome is a genome instability disorder caused by mutations in the DNA helicase RECQL1. J. Clin. Invest. 132, e147301. 10.1172/JCI147301 35025765 PMC8884905

[B2] Andrs M. Hasanova Z. Oravetzova A. Dobrovolna J. Janscak P. (2020). RECQ5: A mysterious helicase at the interface of DNA replication and transcription. Genes (Basel) 11, 232. 10.3390/genes11020232 32098287 PMC7073763

[B3] Cosma J. Russo A. Schino S. Belli M. Mango R. Chiricolo G. (2022). Acute myocardial infarction in a patient with MELAS syndrome: A possible link? Minerva Cardiol. Angiol. 10.23736/S2724-5683.22.06021-5 35767235

[B4] Croteau D. L. Popuri V. Opresko P. L. Bohr V. A. (2014). Human RecQ helicases in DNA repair, recombination, and replication. Annu. Rev. Biochem. 83, 519–552. 10.1146/annurev-biochem-060713-035428 24606147 PMC4586249

[B5] Han F. Pang S. Sun Z. Cui Y. Yan B. (2021). Genetic variants and functional analyses of the ATG16L1 gene promoter in acute myocardial infarction. Front. Genet. 12, 591954. 10.3389/fgene.2021.591954 34220924 PMC8248370

[B6] Haver V. G. Verweij N. Kjekshus J. Fox J. C. Wedel H. Wikstrand J. (2014). The impact of coronary artery disease risk loci on ischemic heart failure severity and prognosis: Association analysis in the COntrolled ROsuvastatin multiNAtional trial in heart failure (CORONA). BMC Med. Genet. 15, 140. 10.1186/s12881-014-0140-3 25528061 PMC4412120

[B7] Hu Y. Raynard S. Sehorn M. G. Lu X. Bussen W. Zheng L. (2007). RECQL5/Recql5 helicase regulates homologous recombination and suppresses tumor formation via disruption of Rad51 presynaptic filaments. Genes Dev. 21, 3073–3084. 10.1101/gad.1609107 18003859 PMC2081974

[B8] Izumikawa K. Yanagida M. Hayano T. Tachikawa H. Komatsu W. Shimamoto A. (2008). Association of human DNA helicase RecQ5beta with RNA polymerase II and its possible role in transcription. Biochem. J. 413, 505–516. 10.1042/BJ20071392 18419580

[B9] Kaier T. E. Alaour B. Marber M. (2021). Cardiac troponin and defining myocardial infarction. Cardiovasc Res. 117, 2203–2215. 10.1093/cvr/cvaa331 33458742 PMC8404461

[B10] Li W. Liu X. Huang C. Liu L. Tan X. Wang X. (2021). The loss-of-function mutation of CETP affects HDLc levels but not ApoA1 in patients with acute myocardial infarction. Nutr. Metab. Cardiovasc Dis. 31, 602–607. 10.1016/j.numecd.2020.10.019 33358712

[B11] Montenegro Junior R. M. Lima G. Fernandes V. O. Montenegro A. Ponte C. M. M. Martins L. V. (2020). Leu124Serfs*26, a novel AGPAT2 mutation in congenital generalized lipodystrophy with early cardiovascular complications. Diabetol. Metab. Syndr. 12, 28. 10.1186/s13098-020-00538-y 32280377 PMC7137278

[B12] Newman J. A. Aitkenhead H. Savitsky P. Gileadi O. (2017). Insights into the RecQ helicase mechanism revealed by the structure of the helicase domain of human RECQL5. Nucleic Acids Res. 45, 4231–4243. 10.1093/nar/gkw1362 28100692 PMC5397160

[B13] Reed G. W. Rossi J. E. Cannon C. P. (2017). Acute myocardial infarction. Lancet 389, 197–210. 10.1016/S0140-6736(16)30677-8 27502078

[B14] Saleh M. Ambrose J. A. (2018). Understanding myocardial infarction. F1000Res 7. 10.12688/f1000research.15096.1 PMC6124376 30228871

[B15] Salwan R. Seth A. (2021). Development of ST-elevation myocardial infarction programs in developing countries: Global challenges and solutions. Interv. Cardiol. Clin. 10, 401–411. 10.1016/j.iccl.2021.03.010 34053626

[B16] Saponaro M. Kantidakis T. Mitter R. Kelly G. P. Heron M. Williams H. (2014). RECQL5 controls transcript elongation and suppresses genome instability associated with transcription stress. Cell 157, 1037–1049. 10.1016/j.cell.2014.03.048 24836610 PMC4032574

[B17] Tavera-Tapia A. De La Hoya M. Calvete O. Martin-Gimeno P. Fernandez V. Macias J. A. (2019). RECQL5: Another DNA helicase potentially involved in hereditary breast cancer susceptibility. Hum. Mutat. 40, 566–577. 10.1002/humu.23732 30817846

[B18] Thakkar M. K. Lee J. Meyer S. Chang V. Y. (2022). RecQ helicase somatic alterations in cancer. Front. Mol. Biosci. 9, 887758. 10.3389/fmolb.2022.887758 35782872 PMC9240438

[B19] Wang J. Xiao Q. Wang L. Wang Y. Wang D. Ding H. (2022). Role of ABCA1 in cardiovascular disease. J. Pers. Med. 12, 1010. 10.3390/jpm12061010 35743794 PMC9225161

[B20] Watany M. M. Nagi Abd-Ellatif R. Ezzelregal Abdeldayem M. El-Sayed El-Horany H. (2022). Association between genetic variations of mitochondrial isocitrate dehydrogenase (IDH2) and acute myocardial infarction. Gene 829, 146497. 10.1016/j.gene.2022.146497 35447240

[B21] Xia H. W. Zhang Z. Q. Yuan J. Niu Q. L. (2021). Human RECQL5 promotes metastasis and resistance to cisplatin in non-small cell lung cancer. Life Sci. 265, 118768. 10.1016/j.lfs.2020.118768 33217443

[B22] Xie X. Zheng Y. Y. Adi D. Yang Y. N. Ma Y. T. Li X. M. (2016). Exome sequencing in a family identifies RECQL5 mutation resulting in early myocardial infarction. Med. Baltim. 95, e2737. 10.1097/MD.0000000000002737 PMC4748938 26844521

[B23] Yu R. Liu L. Chen C. Lin Z. J. Xu J. M. Fan L. L. (2023). A de novo mutation (p.S1419F) of Retinoic acid induced 1 is responsible for a patient with Smith-Magenis syndrome exhibiting schizophrenia. Gene 851, 147028. 10.1016/j.gene.2022.147028 36334618

