# Supplementary figures and images for: Case report: A novel mutation of RecQ-like helicase 5 in a Chinese family with early myocardial infarction, coronary artery disease, and stroke hemiplegia
Source: Front Genet. 2023 Apr 26;14:1146932. doi: 10.3389/fgene.2023.1146932 (PMC10169744; doi:10.3389/fgene.2023.1146932)

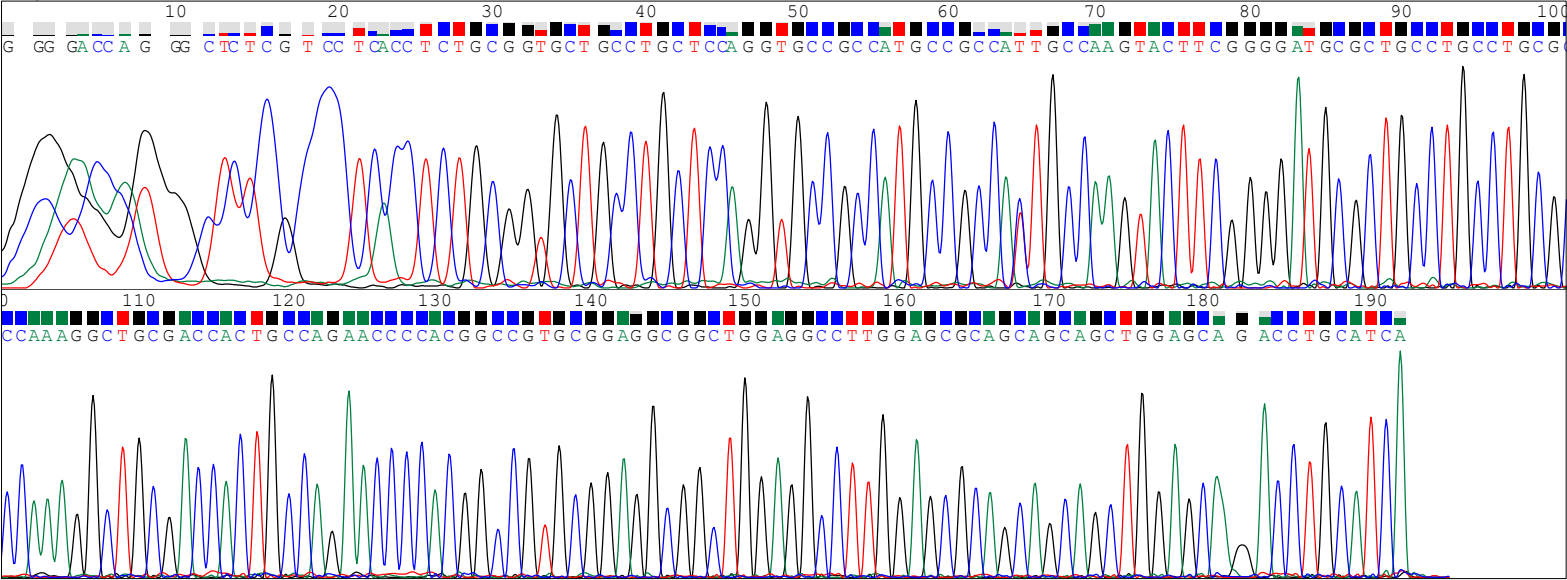

Supplement: Supplementary file 1 [file DataSheet2.PDF]

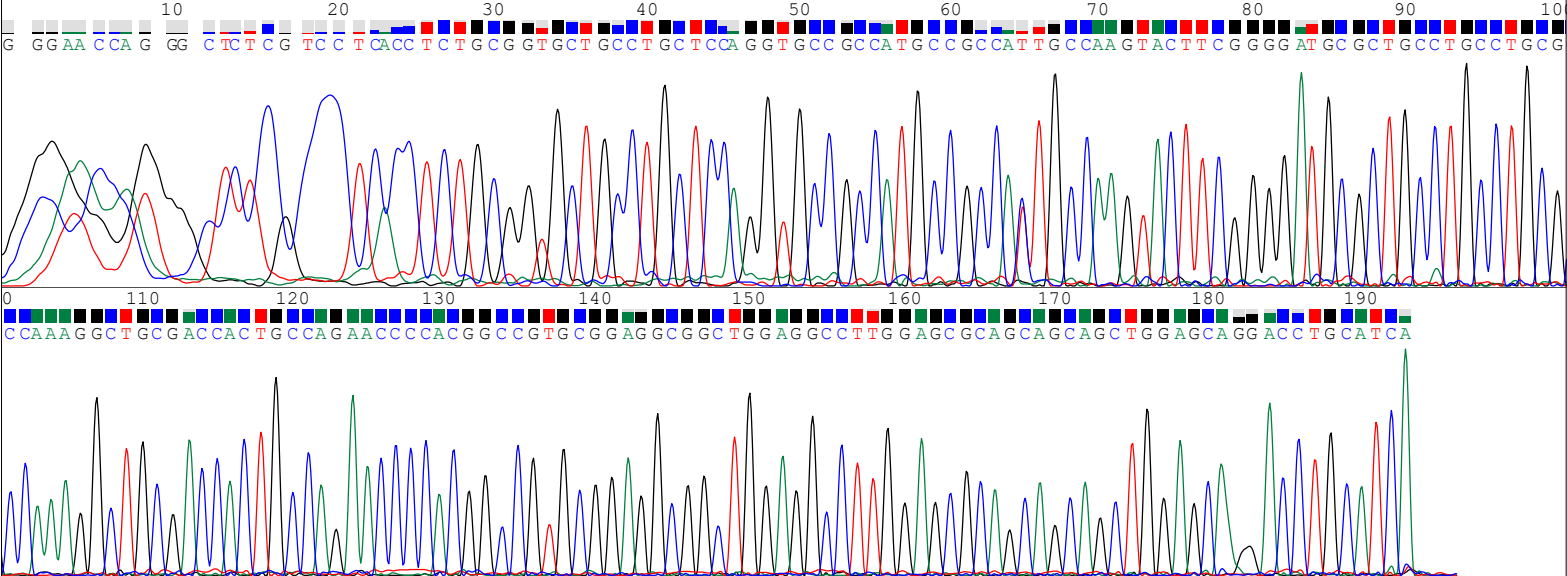

Supplement: Supplementary file 2 [file DataSheet4.PDF]

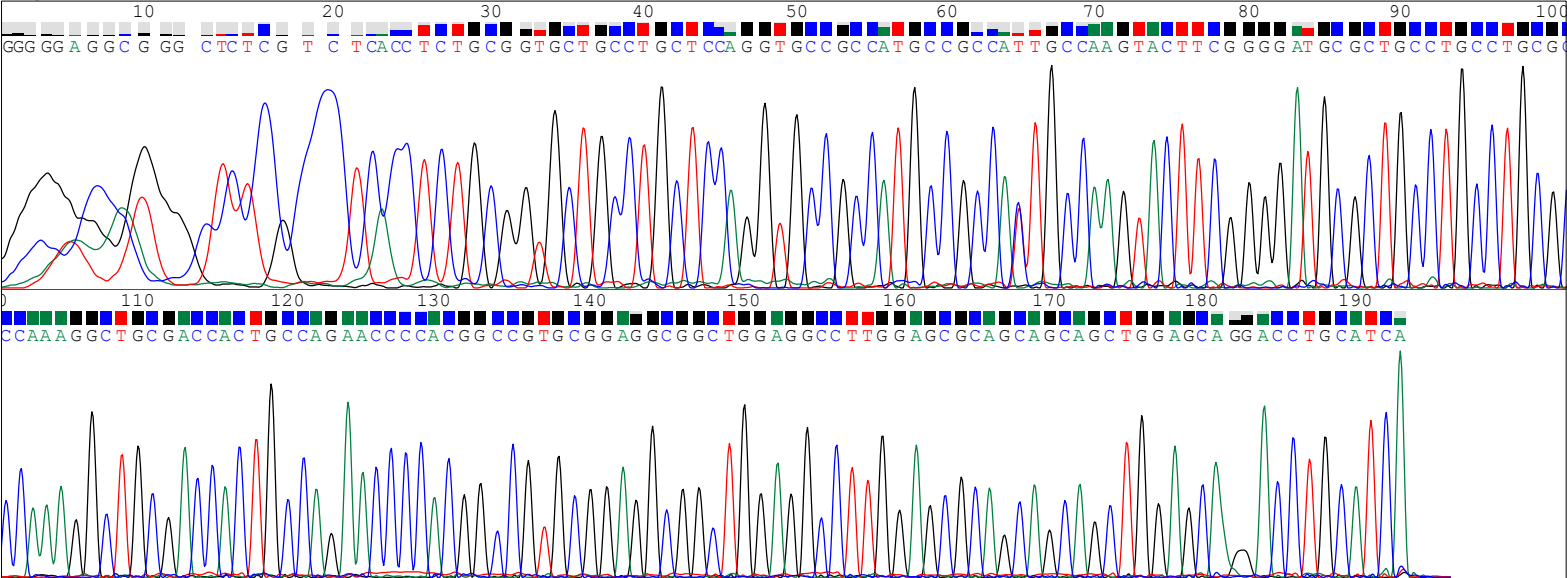

Supplement: Supplementary file 3 [file DataSheet3.PDF]

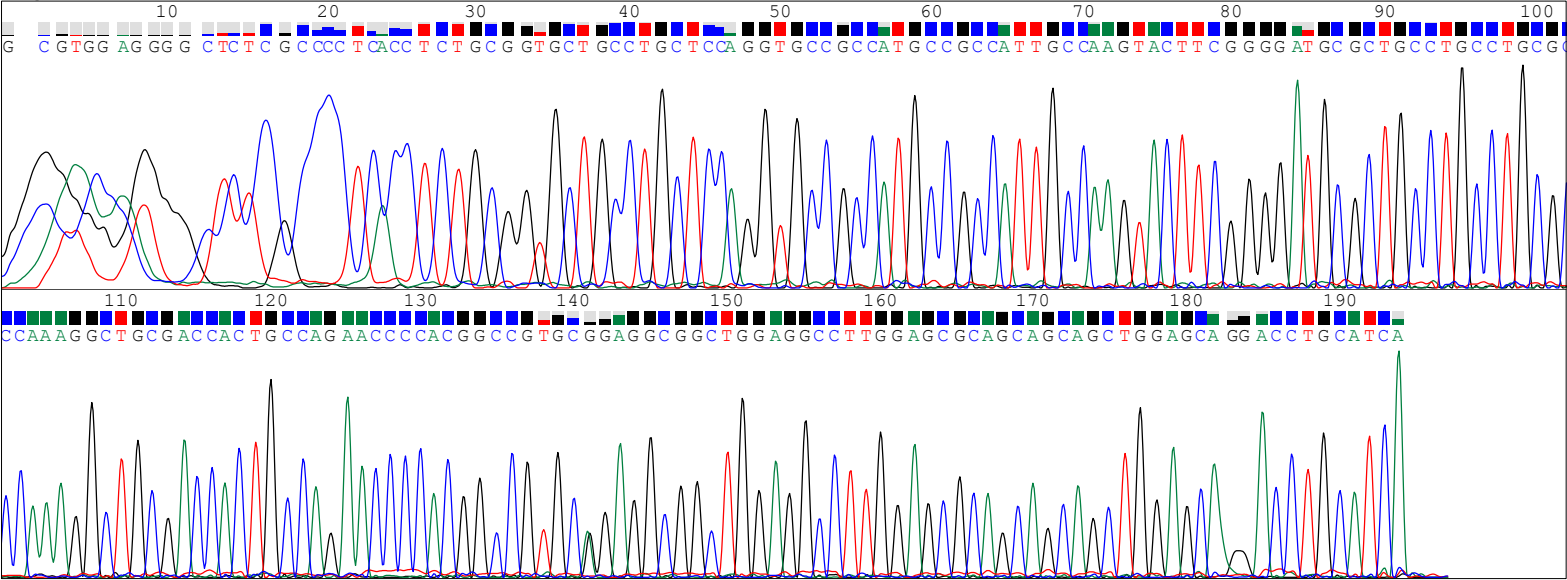

Supplement: Supplementary file 4 [file DataSheet1.PDF]
